# A digital tool for self-reporting cardiovascular risk factors: The RADICAL study

**DOI:** 10.1016/j.ijcrp.2025.200368

**Published:** 2025-01-08

**Authors:** José Ferreira Santos, Inês Castela, Sara Gamboa Madeira, Sofia Furtado, Hugo Vieira Pereira, Diana Teixeira, Hélder Dores

**Affiliations:** a Catolica Medical School, Lisboa, Portugal; b Hospital da Luz Setúbal, Luz Saúde, Portugal; c CHRC, NOVA Medical School, Lisboa, Portugal; d NOVA Medical School, Lisboa, Portugal; e Faculdade de Medicina, Universidade de Lisboa, Lisboa, Portugal; f Hospital da Luz Lisboa, Luz Saúde, Portugal; g CIDEFES – Universidade Lusófona, Lisboa, Portugal; h CoLAB TRIALS, Évora, Portugal

**Keywords:** Cardiovascular, Digital, Prevention, Risk factors, Self-reported

## Abstract

**Aims:**

Cardiovascular diseases remain the leading cause of death worldwide. Risk stratification and early interventions are essential to overcome this reality. The **RADICAL Study***(****R****isk****A****ssessment via****D****igital****I****nput for****C****ardiovascular****A****nd****L****ifestyle Factors)* aimed to evaluate the prevalence of self-reported cardiovascular risk factors in individuals without known cardiovascular disease using a digital tool.

**Methods and results:**

A digital self-reported cardiovascular risk stratification tool, comprising 23 questions about classical and lifestyle cardiovascular risk factors, was completed by 4149 individuals aged 40–69 years (median age 53.0 [47.0; 60.0] years; 78 % women). Among the cardiovascular risk factors, 40.9 % reported hypercholesterolemia, 26.8 % hypertension, 17.3 % smoking, 5.8 % diabetes, 58.4 % physical inactivity, 19.4 % obesity, 33.7 % sleep less than 7 h/night, and 12.1 % had composite dietary risk factors. Most of the participants (89.9 %) referred having at least one of the eight cardiovascular risk factors. Women had 27 % higher odds of having at least one cardiovascular risk factor compared to men (OR = 1.27, 95 % CI [1.00, 1.60]). Participants aged 50–59 years also had higher odds of having at least one CV risk factor compared to those aged 40–49 years (OR = 1.35, 95 % CI [1.07, 1.70]).

**Conclusion:**

The RADICAL Study reveals a high prevalence of cardiovascular risk factors in adults without known cardiovascular disease. Beyond the relevance of traditional risk factors, such as hypercholesterolemia and hypertension, the results regarding physical activity, dietary and sleeping habits are concerning. A self-reported cardiovascular risk identification digital tool could be feasible and help to improve cardiovascular prevention.

## 1 Introduction

Cardiovascular disease (CVD) remains the leading cause of death worldwide, being a real pandemic. In Portugal, approximately 30 % of annual deaths are attributable to CVD, ahead of cancer and respiratory diseases, mostly due to stroke and acute myocardial infarction [1]. In addition, CVD is associated with a significant morbidity and socioeconomic burden, manifested by the high costs of diagnostic exams, treatments, and hospital admissions, as well as the loss of productivity, both of patient and their caregivers [2,3].

Addressing this crucial public health problem is imperative and must start with primary cardiovascular (CV) prevention. Approximately 80 % of CVD related to atherosclerosis can be prevented by improving lifestyle and controlling CV risk factors, being estimated that one out of three deaths can be avoided [4]. However, prevention is often underestimated, with low health literacy being one of the main barriers. This lack of awareness and understanding of the importance of CVD among the general population leads to weak involvement in preventive measures. In this setting, it is urgent to implement measures and strategies to place prevention at the top of the healthcare priorities, developing a culture in society that encourages disease prevention and health promotion. The current evidence is unanimous in demonstrating the widespread poor control of the classical CV risk factors, which are highly prevalent in the general population, such as hypertension, diabetes, dyslipidaemia, obesity, smoking, physical inactivity and dietary risk factors [4,5].

Early detection of these CV risk factors, and risk stratification are essential steps to reduce clinical events. The role of physicians is utmost important for early identification of risk factors, but patients’ involvement could be also crucial to its recognition. In this context, there are several validated and recommended scores for CV risk stratification frequently applied in clinical practice, especially the SCORE2/SCORE2-OP [6]. As these scores must be applied by physicians, or at least by other health professionals, they might not reach individuals who do not regularly use health services, but already have increased CV risk. The development of risk stratification methodologies allowing self-reporting, ideally based on digital tools and without the need for healthcare facilities, could expand the ability to identify individuals at risk. Self-reported CV risk stratification may be particularly suitable for resource-limited environments, while allowing adjustments in the periodicity and type of referral for healthcare, improving the allocation of resources with a potentially favourable socioeconomic impact. In fact, these methodologies, already tested in several contexts, could have positive beneficial effects on global CV health [[7], [8], [9]]. Therefore, the aim of this study was to evaluate the prevalence of self-reported CV risk factors in individuals without known CVD, using a digital tool.

## 2 Methodology

### 2.1 Study design and population recruitment

The **RADICAL Study** *(****R****isk* ***A****ssessment via* ***D****igital* ***I****nput for* ***C****ardiovascular* ***A****nd* ***L****ifestyle Factors)* is an observational, cross-sectional study carried out from October 2023 to March 2024. A digital self-reported CV risk stratification tool, integrating questions about classical and lifestyle CV risk factors, was disseminated and published in social media and other online platforms. The main platform used for recruitment was *Cardio da Vida*® (https://cardiodavida.pt), an online platform whose main objective is to improve the population's literacy and empower professionals in different areas related with CV prevention. The digital self-reported CV risk stratification tool included information for study participants, informed consent, and a questionnaire.

Before enrolment on the study, participants were asked to confirm inclusion and exclusion criteria. Men and women aged 40–69 years from the general population without known CVD, willing and able to provide informed consent were eligible to participate. The only exclusion criterion was a pre-existing history of a CVD, including coronary artery disease; acute myocardial infarction; previous treatment of the coronary arteries by percutaneous intervention or surgery; stroke; transient ischemic attack; peripheral arterial disease (carotid or lower limbs); heart failure; atrial fibrillation; aortic artery aneurysm; valvular heart disease; and other CVD reported in the questionnaire by the participants.

### 2.2 Development and validation of the digital tool

The digital tool integrated 23 questions about both classical and lifestyle CV risk factors, including age, sex, current smoking, body weight, height, blood pressure, lipid profile, diabetes, sleeping habits, dietary habits (fruit and vegetable, red and/or processed meat, and salt intake), and exercise/physical activity. Composite dietary risk factors were defined as the simultaneous occurrence of consuming less than five portions (400 g) of fruits and vegetables daily, consuming at least one portion of red and/or processed meat daily, and adding salt during meal preparation. Body mass index (BMI) was calculated from the reported body weight and height and classified according to the WHO cut-offs used to determine overweight (≥25 kg/m^2^) and obesity (≥30 kg/m^2^) [10].

The validity of a questionnaire is determined by analysing whether it measures what it intends to measure. Evaluation and validation of the questionnaire was carried out by nine invited external experts, with solid clinical and scientific experience in the thematic area of the instrument under analysis. Pre-requisites for the selection of these experts were scientific publications about CV prevention in indexed journals and at least two years of clinical experience. The expert group panel consisted of five cardiologists, two general practitioners’ doctors, one nutritionist and one exercise physiologist.

After presenting the study protocol and signing the informed consent, the expert group received the digital tool and they were invited to answer, anonymously, whether questions were logical and relevant for its purpose. Overall, the expert group agreed on the clarity, content and relevance of the questions presented, and reported an interest in using the questionnaire in future interventions.

### 2.3 Pilot study

After validation of the digital tool by the group of experts, a pilot study was conducted on 40 individuals. The sample size was determined considering the recommendations of Perneger and colleagues [11]. These authors suggest that before carrying out a study with the application of one questionnaire to the targeted population, it is recommended to test the questions on a small sample (around 30 to 50) of participants. The reliability of the questionnaire was assessed using Cronbach's alpha coefficient. A Cronbach's alpha value of 0.7 or higher indicates good internal consistency [12].

The digital tool showed a moderate internal consistency (Cronbach's α = 0.638). Furthermore, the results of this preliminary analysis confirmed the accessibility of the digital tool and the possibility of completing the questionnaire in less than 5 min. Based on this validation, it was considered possible to apply the digital tool to a larger and more representative sample.

### 2.4 Ethics

This study adhered to the principles laid down in the Declaration of Helsinki; on anonymity, voluntary, informed consent and the right to withdraw without any negative consequences. Ethics approval was obtained from the Ethics Committee of NOVA Medical School of the NOVA University of Lisbon (nº 59/2023/CEFCM, June 29, 2023). All participants voluntarily agreed to participate in the study and provided their digital informed consent before answering the questionnaire.

### 2.5 Statistical analysis

Statistical analyses were performed using IBM SPSS for Windows version 29.0 (IBM SPSS, Inc., Chicago, IL). The distribution of continuous variables was tested with Kolmogorov-Smirnov test and absolute values of skewness and kurtosis. Data are presented as means with standard deviation, median with interquartile range [25th–75th percentiles], or numbers with percentages, depending on the nature and distribution of the variables.

Chi-square test was used to determine whether CVD risk factors were associated with sex (men vs. women) and age group (40–49 years vs. 50–59 years vs. 60–69 years). Additionally, odds ratios (OR) with 95 % confidence intervals (CI) were calculated to assess the direction and magnitude of these associations. For testing hypotheses involving continuous variables, nonparametric tests (Mann-Whitney or Kruskal-Wallis) were used as appropriate, considering the normality of the distribution, the number of groups and their independence. All statistical analyses were conducted at a significance level of 0.05, and all tests were two-tailed.

## 3 Results

### 3.1 Population characteristics

**Fig. 1:**
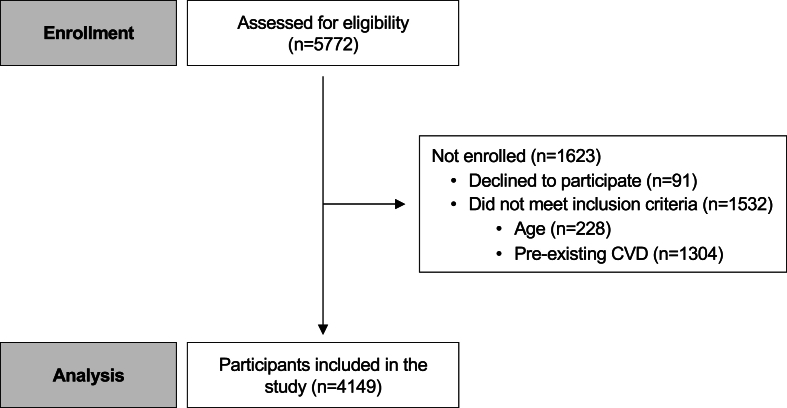
Recruitment and population selection for the RADICAL study.

A total of 4149 participants met inclusion criteria and were included in the study (Fig. 1). The median age was 53.0 [47.0; 60.0] years, and 3234 (77.9 %) individuals were women (Table 1).

**Table 1:** Distribution of reported clinical, anthropometric and biochemical characteristics in study population (n = 4149).

	Overall population (n = 4149)
**Age (years)**, median [IQR]	53.0 [47.0; 60.0]
**Women**, no. (%)	3234 (77.9 %)
**BMI (kg/m**^**2**^**)**, median [IQR]	25.7 [23.1; 29.1]
**Cardiovascular risk factors**, no. (%)	
Hypercholesterolemia	1695 (40.9 %)
Hypertension	1111 (26.8 %)
Diabetes	240 (5.8 %)
Obesity	805 (19.4 %)
Current smoking	716 (17.3 %)
Physical inactivity	2422 (58.4 %)
**Blood pressure and lipid profile**^a^, mean ± SD	
SBP (mmHg)	123.6 ± 14.1
DBP (mmHg)	76.2 ± 10.8
Total cholesterol (mg/dL)	190.4 ± 37.7
HDL cholesterol (mg/dL)	57.4 ± 13.3
Non-HDL cholesterol (mg/dL)	133.0 ± 38.6
**Exercise (hours per week)**^b^, median [IQR]	4.0 [3.0; 6.0]
**Sleep habits (hours per night)**, mean ± SD	6.8 ± 1.0
<7 h per night, no. (%)	1399 (33.7 %)
**Composite dietary risk factor**s^c^**, no. (%)**	501 (12.1 %)
Insufficient fruit and vegetables intake (<5 portions/daily)	2198 (53.0 %)
Red and/or processed meat intake (≥1 portion/daily)	922 (22.2 %)
Adding salt during meals preparation	3251 (78.4 %)
**Multiple cardiovascular risk factor**s^d^, no. (%)	
At least one cardiovascular risk factor	3729 (89.9 %)
Three or more cardiovascular risk factor	1544 (37.2 %)
Five or more cardiovascular risk factor	213 (5.1 %)

Data are presented as mean ± standard deviation or median [interquartile range] and as a number and percentage.

BMI, body mass index; DBP, diastolic blood pressure; HDL, high density lipoprotein; IQR, interquartile range; SBP, systolic blood pressure; SD, standard deviation.

a In participants who reported measurements.

b In participants who regularly exercise.

c Composite dietary risk factors was defined as the simultaneous occurrence of consuming less than five portions (400 g) of fruits and vegetables daily, consuming at least one portion of red and/or processed meat daily, and adding salt during meal preparation.

d Analyse of eight CV risk factors - hypercholesterolemia, hypertension, diabetes, obesity, smoking, sleeping <7 h per night, physical inactivity and composite dietary risk factors (consuming less than five portions (400 g) of fruits and vegetables daily, consuming at least one portion of red and/or processed meat daily, and adding salt during meal preparation).

### 3.2 Prevalence of cardiovascular risk factors

#### 3.2.1 Classical cardiovascular risk factors

Self-reported clinical characteristics of the participants are presented in Table 1. Among the classical CV risk factors, 1695 (40.9 %) participants reported hypercholesterolemia. Of these, 766 indicated they were taking lipid-lowering treatments, corresponding to less than half of them (45.2 %). Out of the total, 1470 (35.4 %) participants had a lipid profile evaluation in the previous six months, presenting a mean total cholesterol level of 190.4 ± 37.7 mg/dL and mean non-HDL cholesterol level of 133.0 ± 38.6 mg/dL. Total cholesterol levels exceeded the normal limit (200 mg/dL) in 13.4 % (556 of 1470) of the participants.

Hypertension was reported by 1111 (26.8 %) individuals, with the majority undergoing treatment (n = 953; 85.8 %). Nearly half of the participants (44.8 %) had measured their blood pressure within the previous 24 h, presenting a mean systolic and diastolic blood pressure of 123.6 ± 14.1 mmHg and of 76.2 ± 10.8 mmHg, respectively. Among those who measured their blood pressure, only 6.0 % had a systolic blood pressure higher than 140 mmHg, and 5.0 % had a diastolic blood pressure higher than 90 mmHg.

The prevalence of diabetes was 5.8 % (n = 240), with the great majority receiving antidiabetic treatments (n = 211; 87.9 %). Participants had a median BMI of 25.7 [23.1; 29.1] kg/m^2^, more than half of the participants (55.0 %) were overweight, while 19.4 % had obesity.

#### 3.2.2 Cardiovascular risk factors related to lifestyle

With respect to lifestyle CV risk factors, 716 (17.3 %) individuals reported currently smoking, and one-third (33.7 %) of the participants reported sleeping less than 7 h a night, with a mean of 6.8 h. A total of 2422 (58.4 %) participants were physically inactive, with a median exercise duration of 4.0 [3.0; 6.0] hours per week among those who exercised (Table 1).

Additionally, more than half of the participants (53.0 %) reported consuming less than five portions of fruits and vegetables daily while 922 (22.2 %) reported consuming at least one portion of red and/or processed meat daily. More than three quarters of the participants (78.4 %) reported adding salt during meal preparation. When we evaluated the prevalence of composite dietary risk factors – defined as the number of participants who simultaneously reported not consuming five portions of fruit and vegetables daily, consuming at least one portion of red and/or processed meat daily, and adding salt during food preparation – we observed a prevalence of 12.1 %.

#### 3.2.3 Clustering of cardiovascular risk factors

Most of the participants (89.9 %) referred having at least one of the eight CV risk factors (hypercholesterolemia, hypertension, diabetes, obesity, smoking, sleeping <7 h per week, physical inactivity or dietary risk factors). One-third (37.2 %) of the participants had three or more risk factors, and a cluster of five or more risk factors was observed in 5.1 % of the participants (Table 1).

### 3.3 Cardiovascular risk factors according to sex and age group

The prevalence of the several CV risk factors differed by sex and age group (Table 2). Hypertension and diabetes were more prevalent in men, whereas hypercholesterolemia and physical inactivity were more common in women. Specifically, men showed 53 % higher odds of having hypertension and 80 % higher odds of having diabetes compared to women (odds ratio, OR = 1.53, 95 % CI [1.31, 1.80] for hypertension; OR = 1.80, 95 % CI [1.36, 2.38] for diabetes). In contrast, men had 15 % lower odds of having hypercholesterolemia (OR = 0.85, 95 % CI [0.73, 0.98]), and were 1.76 times more likely to be physically active than women (OR = 1.76, 95 % CI [1.51, 2.04]). Only obesity, smoking and sleeping habits did not reveal any association with sex. Regarding dietary habits, men had a higher prevalence of inadequate fruit and vegetable intake, as well as higher consumption of red and/or processed meat, while women had a higher prevalence of adding salt during meal preparation. However, despite differences observed in questions about specific food groups, the prevalence of composite dietary risk factors was similar between men and women (13.9 % vs. 11.5 %, *P* = 0.054).

**Table 2:** Distribution of cardiovascular risk factors by sex and age group.

	Sex		*P-value*	Age, years			*P-value*
	Male (n = 907)	Female (n = 3234)		40-49 (n = 1476)	50-59 (n = 1582)	60-69 (n = 1091)	
**BMI (kg/m**^**2**^**)**, median [IQR]	26.5 [24.3; 29.3]	25.4 [22.7; 28.9]	**<0.001∗∗**	25.4 [22.6; 28.9]	25.8 [23.3; 29.1]	25.9 [23.6; 29.1]	**0.001∗∗**
**Cardiovascular risk factors**, no. (%)							
Hypercholesterolemia	342 (37.7 %)	1349 (41.7 %)	**0.030∗**	496 (33.6 %)	686 (43.4 %)	513 (47.0 %)	**<0.001∗∗**
Hypertension	305 (33.6 %)	804 (24.9 %)	**<0.001∗∗**	235 (15.9 %)	437 (27.6 %)	439 (40.2 %)	**<0.001∗∗**
Diabetes	78 (8.6 %)	161 (5.0 %)	**<0.001∗∗**	39 (2.6 %)	84 (5.3 %)	117 (10.7 %)	**<0.001∗∗**
Obesity	174 (19.2 %)	630 (19.5 %)	0.771	278 (18.8 %)	319 (20.2 %)	208 (19.1 %)	0.586
Current Smoking	164 (18.1 %)	550 (17.0 %)	0.449	264 (17.9 %)	279 (17.6 %)	173 (15.9 %)	0.356
Physical inactivity	476 (52.5 %)	1985 (61.4 %)	**<0.001∗∗**	931 (63.1 %)	929 (58.7 %)	562 (51.5 %)	**<0.001∗∗**
**Sleep habits**, no. (%)							
<7 h per night	324 (35.7 %)	1073 (33.2 %)	0.152	476 (32.2 %)	548 (34.6 %)	375 (34.4 %)	0.327
**Composite dietary risk factor**s^a^, no. (%)	126 (13.9 %)	373 (11.5 %)	0.054	244 (16.5 %)	176 (11.1 %)	81 (7.4 %)	**<0.001∗∗**
Insufficient fruit and vegetables intake (<5 portions/daily)	510 (56.2 %)	1684 (52.1 %)	**0.027∗**	866 (58.7 %)	839 (53.0 %)	493 (45.2 %)	**<0.001∗∗**
Red and/or processed meat intake (≥1 portion/daily)	285 (31.4 %)	634 (19.6 %)	**<0.001∗∗**	436 (29.5 %)	321 (20.3 %)	165 (15.1 %)	**<0.001∗∗**
Adding salt during meals preparation	560 (61.7 %)	2686 (83.1 %)	**<0.001∗∗**	1152 (78.0 %)	1265 (80.0 %)	834 (76.4 %)	0.089
**Multiple cardiovascular risk factor**s^b^, no. (%)							
At least one cardiovascular risk factor	799 (88.1 %)	2922 (90.4 %)	**0.046∗**	1302 (88.2 %)	1439 (91.0 %)	988 (90.6 %)	**0.029∗**

Data are presented as median [interquartile range] and as a number and percentage. Differences between groups were determined by Mann-Whitney, Kruskal-Wallis test or chi-squared test, according to the distribution of variables and the number and independency of groups compared. Differences were considered statistically significant when ∗*P* < 0.05 and ∗∗*P* < 0.001.

BMI, body mass index; IQR, interquartile range.

a Composite dietary risk factors was defined as the simultaneous occurrence of consuming less than five portions (400 g) of fruits and vegetables daily, consuming at least one portion of red and/or processed meat daily, and adding salt during meal preparation.

b Analyse of eight CV risk factors - hypercholesterolemia, hypertension, diabetes, obesity, smoking, sleeping <7 h per night, physical inactivity and composite dietary risk factors (consuming less than five portions (400 g) of fruits and vegetables daily, consuming at least one portion of red and/or processed meat daily, and adding salt during meal preparation).

As expected, the prevalence of hypercholesterolemia, hypertension, diabetes and physical inactivity increased with age. Participants aged 40–49 years had 34 % and 43 % lower odds of having hypercholesterolemia compared to those aged 50–59 years (OR = 0.66, 95 % CI [0.57, 0.77]) and those aged 60–69 years (OR = 0.57, 95 % CI [0.49, 0.67]), respectively. The proportion of participants with hypercholesterolemia was similar in the older age groups (50–59 vs. 60–69 years). Participants aged 40–49 years also had lower odds of having hypertension, with 50 % and 72 % lower odds compared to those aged 50–59 years (OR = 0.50, 95 % CI [0.42, 0.59]) and those aged 60–69 years (OR = 0.28, 95 % CI [0.23, 0.34]), respectively. The odds of having hypertension were 0.57 times lower in participants aged 50–59 years than those older (OR = 0.57, 95 % CI [0.48, 0.67]). Furthermore, participants aged 40–49 years had significantly lower odds of having diabetes compared to those aged 50–59 years (OR = 0.48, 95 % CI [0.33, 0.71]) and those aged 60–69 years (OR = 0.23, 95 % CI [0.16, 0.33]). Participants aged 50–59 years were 53 % less likely to have diabetes than those aged 60–69 (OR = 0.47, 95 % CI [0.35, 0.63]). Lastly, participants aged 40–49 and 50–59 years were 38 % and 25 % less likely to be physically active compared to those aged 60–69 years (OR = 0.62, 95 % CI [0.53, 0.73] for 40–49 years; OR = 0.75, 95 % CI [0.64, 0.87] for 50–59 years), respectively. The odds of being physically active were 17 % lower in participants aged 40–49 years than those aged 50–59 years (OR = 0.83, 95 % CI [0.72, 0.96]).

Similar to the analysis stratified by sex, the prevalence of obesity, smoking, and sleeping habits was consistent across age groups. Moreover, dietary habits appeared to improve with age, as younger individuals had a higher prevalence of low fruit and vegetable intake and daily consumption of red and/or processed meat. There were no significant differences in the prevalence of adding salt during meal preparation between age groups. Older participants (aged 60–69 years) had significantly lower odds of having composite dietary risk factors compared to those aged 40–49 years (OR = 0.41, 95 % CI [0.31, 0.53]) and those aged 50–59 years (OR = 0.64, 95 % CI [0.49, 0.84]). Participants aged 50–59 years were 37 % less likely to have composite dietary risk factors than those aged 40–49 (OR = 0.63, 95 % CI [0.51, 0.78]).

#### 3.3.1 Clustering of cardiovascular risk factors

**Fig. 2:**
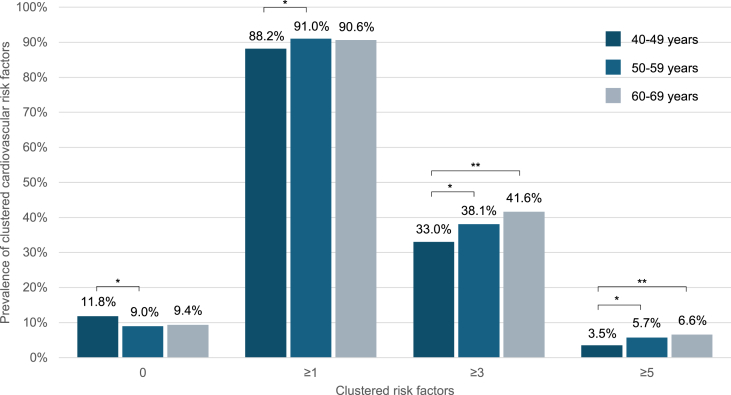
Distribution of cardiovascular risk factors cluster by age group. Data are presented as percentage. Differences between groups were determined by chi-squared test and it were considered statistically significant when ∗*P* < 0.05 and ∗∗*P* < 0.001.

The presence of multiple CV risk factors was also associated with sex and age. Women had a higher prevalence of having at least one CV risk factor, with 27 % higher odds compared to men (OR = 1.27, 95 % CI [1.00, 1.60]). Participants aged 50–59 years had 35 % higher odds of having at least one CV risk factor compared to those aged 40–49 years (OR = 1.35, 95 % CI [1.07, 1.70]). The prevalence of having at least one CV factor was similar between participants aged 40–49 or 50–59 years and those aged 60–69 years (Fig. 2).

## 4 Discussion

The RADICAL study shows a high prevalence of several modifiable CV risk factors in individuals without known CVD. Most individuals had at least one CV risk factor, being particularly relevant the prevalence of hypercholesterolemia, physical inactivity, hypertension, and overweight/obesity. Dietary risk factors and sleep habits were also important findings identified in this analysis.

Self-reporting of CV risk factors using a digital tool seems feasible and could be useful for risk stratification, widening the screening of individuals at increased risk, improving the management of these risk factors, and the referral for evaluation by healthcare professionals. Our data also highlight the necessity of targeted interventions and integrated strategies for early detection and treatment of modifiable CV risk factors.

Even with some regional variations, the overall prevalence of modifiable CV risk factors among European countries is high and in accordance with the burden of CVD [13]. Although the novel methodology used to report CV risk factors in this study, its overall prevalence in the Portuguese population aligns with already known and published data. For example, in the AMALIA study, which used a community questionnaire applied through a household approach, the self-reported prevalence of hypertension was 23.5 %, hypercholesterolemia 19.7 %, diabetes 8.9 %, smoking 16.3 %, overweight/obesity 51.6 %, and physical inactivity 65.3 % [14]. Also the prevalence of inadequate consumption of fruits and vegetables, as well as red and processed meats, was consistent with that observed in the National Food, Nutrition and Physical Activity Survey of the Portuguese general population (2015–2016) [15]. This consistency may enhance the perceived robustness of the digital tool used in this study, as despite relying on self-reported data, it demonstrates its ability to adequately assess CV risk factors.

To decrease this current impact of CVD, early identification of risk factors and implementing strategies to improve CV prevention, especially based on lifestyle modification, is essential. Improving health literacy is crucial for this purpose, through sharing clear and simple messages about recognizing risk factors, understanding their implications, and adopting basic preventive measures. The first step to optimize CV prevention is risk stratification, which allows for the implementation of preventive and therapeutic interventions tailored to each risk class [4,5]. The Systematic Coronary Risk Evaluation 2 (SCORE2) and SCORE2-OP are currently recommended by the European Society of Cardiology, estimating the risk of fatal and non-fatal CV events at 10 years in individuals aged between 40 and 69 years and 70 or over, respectively. These scores encompass multiple variables with proven associations with CVD development, including age, sex, systolic blood pressure, non-HDL cholesterol and smoking, and are applied to apparently healthy individuals [4]. Despite the unquestionable usefulness of these models, some controversial aspects persist, such as their scope and widespread practical applicability in the population setting. At the physician level, some barriers also limit the implementation of CV risk calculation scores.

Current risk scores rely on a set of clinical features and laboratory measures that typically require the presence of a physician, which limit and reduce the ability to evaluate many individuals without access to face-to-face visits. In this setting, there is a growing number of self-assessment tools designed for risk prediction across several health conditions. These tools are mainly intended to alert, educate, modify lifestyle behaviours, or directly refer high-risk individuals to healthcare professionals for further evaluation. In the actual era, characterised by the widespread availability of digital tools aimed to facilitate healthcare delivery, involving patients in their own risk stratification, is increasingly relevant. Internet and smartphone use has exponentially grown in the past decade, opening the possibility that these increasingly prevalent technological tools could improve health. Digital health interventions, using modalities such as telemedicine, web-based strategies, email, mobile applications, text messaging, and monitoring sensors, can potentially shift healthcare from the traditional physical medical institutions to a more individualised and accessible model of care [16].

Self-reporting risk factors using digital tools can provide a reliable and accurate estimation of CV risk. These tools must be simple, provide a quick estimation and clear individualised recommendations [11]. The tool used in our study meets these criteria. Insights from the Minnesota Heart Survey showed that self-reported knowledge of risk may be useful for identifying individuals at low CV risk, but the authors emphasize that such self-reporting should always be followed by clinical evaluation if symptoms or history suggestive of CVD [9]. The DiCAVA, a 10-year CVD risk model based on machine learning, demonstrated a very good predictive capacity and feasible utility in remote settings where cholesterol and blood pressure measurements are not always convenient. This highlight that even the most well-established predictors are not always essential [8]. Another novel, simple risk score showed to be an easy-to-use tool to predict CV events in adults from self-reported information without the need for laboratory or physical examination data. This risk score included 6 items and had comparable predictive performance to the guideline-recommended atherosclerotic CVD risk score but relied solely on self-reported information [17]. Self-screening for assessing CV risk showed similar results to the standard methods, with high clinical performance to rule out intermediate or high CV risk [7]. Even in elderly individuals, the use of digital tools seems to be feasible, as evidenced by a community-based cross-sectional survey conducted among Malaysian older adults (7117 individuals aged ≥50 years), which used a standardised structured questionnaire to evaluate the prevalence of self-reported CV risk factors [18].

Beyond the role of self-reporting risk factors and other characteristics, digital tools have also been used in interventions to control risk factors and decrease CVD risk. A meta-analysis aiming to assess the benefit of digital health interventions on CVD outcomes (CVD events, all-cause mortality, hospitalizations) and risk factors, showed that these strategies can overall reduce CVD outcomes (nearly 40 %) and have a positive impact on risk factors, versus usual care. These benefits were largely driven by improvements in outcomes among higher risk individuals, including in those targeting secondary CVD prevention [19]. Another meta-analysis suggests that specific digital health interventions, namely automated email messages, may improve CVD risk outcomes. However, as the evidence was inconclusive for some of the interventions analysed, further research into specific digital modalities is required, including newer technologies, such as wearable tools [20]. The benefits of digital interventions have been shown in the control of specific CV risk factors, including smoking cessation, exercise adherence, weight loss, diabetes, and hypertension self-management [[21], [22], [23], [24], [25]].

It is essential that healthcare providers consider digital health interventions to promote CVD prevention. Further research is needed to explore more recent and interactive tools, such as those using artificial intelligence, improving the interaction with the user, and integrating personalised feedback from healthcare professionals. Independently of the adopted methodology, and as deeply recognised, the risk factors evaluated in our study are essential components that should be incorporated to assess and improve CV health [26].

The present study has some limitations. Self-assessment of CV risk factors, without previous specific education and explanation could have led to misreporting. Otherwise, the voluntary participation and the use of social media and digital tools could influence the characteristics of the participants, as for example the higher number of women. As some analysis already revealed, some subjects are not aware of, or under report, the presence of CV risk factors. The potential discrepancies between self-reported and objective presence of CV risk factors reveal that some caution must exist when using self-reported data [27]. This self-misclassification can lead to inappropriate referral to healthcare for additional evaluation and lead to unnecessary costs or treatments. To mitigate this fact, the reported answers by the participants in this study and the used digital tool should be validated. In this context, development of educational interventions is essential to improve personal knowledge about CV risk factors. Some important determinants of CV risk were not evaluated but could be important to for the overall risk evaluation, especially socioeconomic factors, and regional distribution.

The RADICAL Study shows a high prevalence of modifiable CV risk factors in adults without known CV disease. Beyond the relevance of traditional risk factors, such as hypercholesterolemia, overweight/obesity and hypertension, the results regarding physical activity, dietary and sleeping habits are concerning. A self-reported identification of CV risk factors using a digital tool could be feasible and help to improve CV prevention. Further research is needed to explore the application of more recent digital technology on CV risk stratification and interventions to reduce the burden of CVD.

## CRediT authorship contribution statement

**José Ferreira Santos:** Writing – review & editing, Writing – original draft, Visualization, Validation, Supervision, Software, Resources, Project administration, Methodology, Investigation, Formal analysis, Data curation, Conceptualization. **Inês Castela:** Writing – review & editing, Writing – original draft, Visualization, Methodology, Investigation. **Sara Gamboa Madeira:** Writing – review & editing, Validation, Methodology, Investigation, Conceptualization. **Sofia Furtado:** Writing – review & editing, Validation, Supervision, Investigation, Conceptualization. **Hugo Vieira Pereira:** Writing – review & editing, Validation, Conceptualization. **Diana Teixeira:** Writing – review & editing, Writing – original draft, Visualization, Validation, Supervision, Project administration, Methodology, Investigation, Formal analysis, Data curation, Conceptualization. **Hélder Dores:** Writing – review & editing, Writing – original draft, Visualization, Validation, Supervision, Software, Resources, Project administration, Methodology, Investigation, Funding acquisition, Formal analysis, Data curation, Conceptualization.

## Declaration of competing interest

No conflicts of interest, financial or otherwise, are declared by the authors.
